# Contribution of Epstein–Barr Virus Latent Proteins to the Pathogenesis of Classical Hodgkin Lymphoma

**DOI:** 10.3390/pathogens7030059

**Published:** 2018-06-27

**Authors:** Katerina Vrzalikova, Taofik Sunmonu, Gary Reynolds, Paul Murray

**Affiliations:** 1 Institute for Cancer and Genomic Medicine, University of Birmingham, Birmingham B15 2TT, UK; taosun77@gmail.com; 2 Institute of Immunology and Immunotherapy, University of Birmingham, Birmingham B15 2TT, UK; g.m.reynolds@bham.ac.uk; 3 Department of Clinical and Molecular Pathology, Institute of Molecular and Translational Medicine, Faculty of Medicine and Dentistry, Palacky University, 775 15 Olomouc, Czech Republic

**Keywords:** Epstein–Barr virus, Hodgkin lymphoma, latency, B cells

## Abstract

Pathogenic viruses have evolved to manipulate the host cell utilising a variety of strategies including expression of viral proteins to hijack or mimic the activity of cellular functions. DNA tumour viruses often establish latent infection in which no new virions are produced, characterized by the expression of a restricted repertoire of so-called latent viral genes. These latent genes serve to remodel cellular functions to ensure survival of the virus within host cells, often for the lifetime of the infected individual. However, under certain circumstances, virus infection may contribute to transformation of the host cell; this event is not a usual outcome of infection. Here, we review how the Epstein–Barr virus (EBV), the prototypic oncogenic human virus, modulates host cell functions, with a focus on the role of the EBV latent genes in classical Hodgkin lymphoma.

## 1. EBV Is a Transforming B Lymphotropic Virus

EBV is a gamma-herpesvirus that persists asymptomatically in the majority of the world’s adult population through its ability to colonise the B-cell system. Primary infection is usually asymptomatic, in most cases occurring early in life; only a few individuals develop symptoms if infection occurs later, resulting in infectious mononucleosis (IM).

EBV-infected B cells present in the blood of asymptomatically infected individuals when cultured can grow out as EBV-transformed cell lines, these are referred to as lymphoblastoid cell lines (LCL). This can happen only if T cells are removed or inhibited, for example with cyclosporin A, underscoring the requirement for control over the virus by T cells in people [1]. LCL can be made by infecting B cells with EBV in vitro. The EBV genes required for transformation of B cells include ‘latent’ genes, that is, those present in latency, when no virions are made. Another phase of infection can occur when the virus undergoes the replicative cycle in which new virions are assembled and released; the so-called ‘lytic’ genes are required for this process. The latent genes encode six Epstein–Barr nuclear antigens (EBNAs 1, 2, 3A, 3B, 3C and EBNA-LP), the latent membrane proteins (LMP1, LMP2A and LMP2B), two noncoding Epstein–Barr-encoded RNAs (EBER1 and EBER2), and viral miRNA [2,3]. LCL express all known EBV latent genes; this form of infection is known as latency III. Only EBNA2, EBNA3A, EBNA3C and LMP1 have been shown to be essential for the in-vitro transformation of B cells [4], although EBNA-LP was recently shown to be required for the transformation of naïve B cells [5].

## 2. Asymptomatic Infection of B Cells

EBV is a persistent virus, residing in memory B cells for the life of the infected host [6]. There are several models to explain this. In the so-called germinal centre (GC) model, EBV infection of naïve B cells initiates their expansion through proliferation, the resulting immortalized cells expressing the latency III programme. At some point these EBV-infected B cells acquire a GC phenotype, although it is not certain if this occurs in the context of a GC structure. Here the cells express latency II, an alternative form of latency, characterised by expression of EBNA1, LMP1 and LMP2 (of which there are two isoforms; A and B). However, unlike latency III, the other EBNAs are not expressed [7]. LMP1 and LMP2A are CD40 and B-cell receptor (BCR) mimics, respectively; together they are responsible for providing the necessary cues for the post-GC differentiation of the EBV-infected cells [8,9]. The function of LMP2B is poorly understood but it may play a role in negatively regulating LMP2A’s function [10]. After differentiation to memory B cells, the virus expresses no viral proteins; this is known as latency 0 and occurs to prevent detection of the infected cell by the host’s immune response. There is only occasional EBNA1 expression which is required by the virus to ensure episome segregation when the B cells proliferate (this phase is known as latency I) [7]. Alternatively, the virus-infected B cell can differentiate into a plasma cell; this process switches on the virus replicative cycle and provides the means for the virus to make new virions, which can be shed into the oral cavity from trafficking plasma cells in the oral lymphoid tissues [11].

B-cell lymphomas, including Hodgkin lymphoma (HL), Burkitt lymphoma (BL) and diffuse large B-cell lymphoma (DLBCL), can result when these finely tuned interactions between the virus and the host B cell go wrong. EBV-associated HL is the focus of this review.

## 3. Hodgkin Lymphoma (HL)

The hallmark of HL is the existence of a tumour microenvironment (TME) rich in nonmalignant T- and B-lymphocytes and other cell types which surround a minor population of malignant Hodgkin/Reed–Sternberg (HRS) cells. There is evidence that crosstalk between HRS cells and these nonmalignant cells of the TME provides essential signals to HRS cells for their growth and survival. As we shall see later, these TME interactions also promote the escape of HRS cells from an EBV-specific immune response [12].

HL is divided into two major types; classical HL (cHL) and nodular lymphocyte predominant HL (NLPHL). cHL is further separated into four subtypes; nodular sclerosis, mixed cellularity, lymphocyte depletion and lymphocyte-rich classical HL. NLPHL and cHL are morphologically and immunophenotypically distinct; the tumour cells of NLPHL, known as lymphocytic and histiocytic (L&H) cells, are often negative for classical HL markers (e.g., CD15, CD30), but express B-cell markers, including CD20 and CD19, which are absent from the HRS cells of cHL [13].

### 3.1. B-Cell Origin of Hodgkin Lymphoma

The tumour cells of HL have clonally rearranged immunoglobulin genes and somatic IGH mutations in the variable (V) region, indicating their GC or post-GC origin [14,15,16]. However, there is one major difference between cHL and NLPHL in this respect; while the L&H cells of NLPHL show intraclonal V gene diversity indicating that they are derived from differentiating GC B cells [15,17], around one quarter of cHL contain HRS cells bearing nonfunctional mutations in the IGVH genes. Cells bearing these damaging mutations should die by apoptosis (because the rescue of B cells from apoptosis in the GC requires a functional B-cell receptor). For this reason, transforming events occurring in cHL must include the rescue of progenitors from apoptosis [14,15,16].

As indicated above, the HRS cells of cHL do not express typical B-cell markers. In fact, there is global downregulation of B-cell lineage markers accompanied by the overexpression of markers of other haematopoietic cell types including T cells, NK cells and myeloid cells [18,19,20,21,22,23]. The loss of B-cell identity results in part from the disruption of networks of transcription factors including PAX5, early B-cell factor 1 (EBF1) and TCF3/E2A that regulate normal B-cell development and differentiation [22,23,24,25,26,27,28,29,30,31,32,33,34,35].

### 3.2. Deregulated Cellular Signalling in Classical Hodgkin Lymphoma

HRS cells show constitutive activation of canonical and noncanonical nuclear factor kappa B (NF-κB) signalling [24]. Expression of different TNF receptors, such as CD30, CD40, TACI, BCMA and RANK, can induce NF-κB activation in HRS cells [25,26,27,28], following ligation by factors present in the TME [29,30]. IRF5 is also aberrantly activated in HRS cells and co-operates with NF-κB [31]. Notch signalling can also induce NF-κB activation [32].

Activation of the NF-κB pathway can also result from genomic alterations, including c-REL amplifications [33,34,35,36], mutations in IκB inhibitors [37,38,39,40,41], overexpression of BCL3 [42,43] and mutations/deletions in TNFAIP3/A20 [36,44,45]. The noncanonical NF-κB pathway also contributes to the survival of HL cell lines through the stabilisation of the NF-κB inducing kinase (NIK) protein [46]. Chromosomal gains of NIK are reported in primary cHL [36,46,47].

HRS cells can secrete different cytokines, including IL-3, IL-7, IL-9 and IL-13 [48,49,50,51,52], and IL-21 [53,54], which activate JAK/STAT signalling [54,55,56,57]. JAK/STAT signalling can also be dysregulated through JAK2 amplification or loss-of-function mutations of SOCS1 and PTPN1/PTPB1 [58,59,60,61,62].

HRS cells also show deregulated AP-1 signalling [63,64], and we and others have shown that phosphatidylinositide-3-kinase (PI3-K) signalling is constitutively activated in cHL [65,66]. Recently, we have shown that a feed-forward signalling loop driven by the sphingosine-1-phosphate (S1P) receptor-1 (S1PR1) is responsible, at least in part, for the aberrant activation of PI3-K signalling in cHL [67]. Aberrant overexpression and/or activation of different receptor tyrosine kinases (RTK) is also a feature of HRS cells. Two collagen-binding RTK, DDR1 and DDR2, are of particular interest given that many cases of cHL show prominent collagen deposition [68,69,70]. We have shown that the activation of DDR1 by collagen is important for the survival of HRS cells [71].

### 3.3. EBV Is Involved in the Pathogenesis of a Subset of Classical Hodgkin Lymphoma

Elevated antibody titres to the EBV viral capsid antigen (VCA) were initially observed in the blood of cHL patients [72], and appear in people several years prior to the development of cHL [73]. A significantly increased risk of EBV-positive, but not of EBV-negative, cHL is reported for individuals who have had IM [74,75,76,77]. In 1985, the anticomplement immunofluorescence assay was used to detect an EBV protein in HRS cell nuclei; later this protein was designated EBNA1 [78]. Subsequently, EBV DNA was detected in around one-quarter of whole HL biopsies by Southern blot [79]. Later, EBV genomic DNA and EBER expression were detected in HRS cells using sensitive in-situ hybridisation assays [80,81,82]. EBV has also been shown to be present in HRS cells throughout the course of disease and in multiple sites of disease [83]. As with many other EBV-associated cancers, viral genomes in HRS cells exist in monoclonal form; thus it is very likely that all EBV-infected tumour cells arose from a single infected progenitor [80]. It must be noted that there are those who still maintain that EBV is simply a passenger in the process of lymphomagenesis. However, were this the case then the frequency of EBV-positive lymphomas in the population would approximate the frequency of EBV-infected B cells in individuals, that is, 1 in 10^6^, and not the 1 in 3 observed for cHL in the West (see below).

EBV rates in cHL vary depending upon age, gender, histological subtype, ethnicity and country of residence [84,85]. EBV is more commonly found in the tumour cells of cHL patients from underdeveloped countries, and less frequently in cHL patients from the West [86,87]. In Europe and North America, EBV rates in cHL are higher in older people and in children <10 years old, but much lower in young adults [88,89], but also vary by ethnicity and social class [84,90]. These data have been used to suggest that cHL actually comprises three different diseases: (1) childhood cHL, which is EBV-positive and often of mixed cellularity type; (2) young-adult cHL (EBV-negative, nodular sclerosis type); and (3) cHL of older adults (EBV-positive, and usually of mixed cellularity type) [88]. It is entirely likely that EBV’s role in disease pathogenesis differs within these age groups. Furthermore, there is almost certainly a contribution from disorders of EBV-specific immune function. For example, the higher rates of EBV-positive cHL in the elderly have been attributed to declining EBV-specific immunity associated with advancing age [88].

### 3.4. Contribution of EBV Latent Genes to the Pathogenesis of Classical Hodgkin Lymphoma

**Figure 1:**
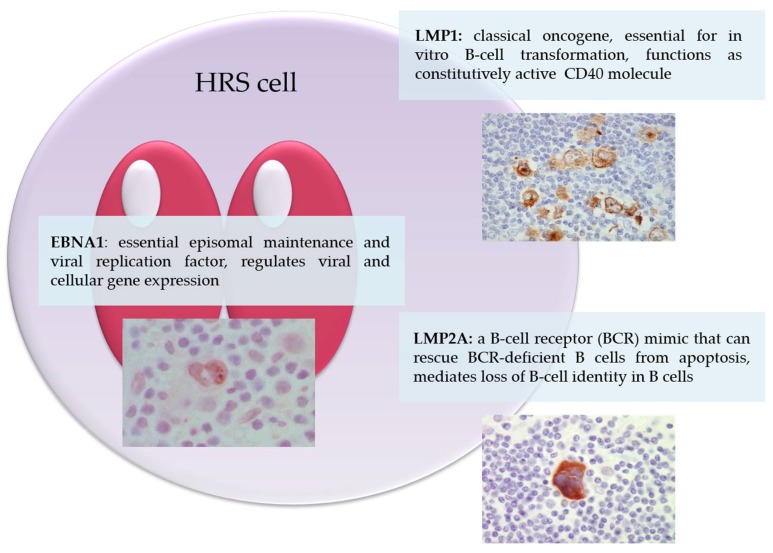
Epstein–Barr virus latent proteins expressed in Hodgkin/Reed–Sternberg cells. Depicted is a Hodgkin/Reed–Sternberg cell with indicated functions of the viral EBNA1, LMP1 and LMP2A proteins.

The EBV genome in HRS cells expresses a latency II pattern including EBNA1 and the latent membrane proteins as well as the viral RNA (Epstein–Barr-encoded RNAs, EBER1 and EBER2, and BART miRNAs). The contribution of virally encoded RNA to the pathogenesis of EBV-associated cHL remains very poorly understood. We now provide a description of the role of EBNA1, LMP1 and LMP2A in the pathogenesis of cHL (Figure 1).

#### 3.4.1. Epstein–Barr Virus Nuclear Antigen-1 (EBNA1)

The maintenance of EBV episomes in infected cells requires EBNA1 which acts both as a viral replication factor and as a tether binding the viral genome to the chromosomes and ensuring faithful segregation of episomes during mitosis [91].

EBNA1 is also reported as a regulator of the transcription of both viral and cellular genes [92,93,94]. Several studies suggest that EBNA1 can directly influence the growth and survival of B cells. For example, knock-down of EBNA1 reduced the survival of BL cells carrying viral episomes but did not cause loss of virus genomes, and also reduced the survival of Namalwa cells, which carry EBV as an integrated genome [95]. EBNA1 also inhibited p53-mediated apoptosis in response to UV irradiation, whereas mutant EBNA1 offered no such protection [95,96]. EBNA1 also enhances the formation of cHL xenografts in NOD-SCID mice [97]. EBNA1 can inhibit TGFβ signalling, in part through increasing the turnover of SMAD2 [98,99], and can promote the growth and survival of cHL cells by downregulating the TGFβ target gene, PTPRK [98]. EBNA1 was also shown to upregulate expression of the chemokine CCL20 in HRS cells, which in turn promoted the migration of regulatory T cells [100]. Thus, EBNA1 might contribute to immune evasion of EBV-infected HRS cells. EBNA1 can also induce B-cell lymphomas in transgenic mice [101,102], although these results were not reproduced in another independent transgenic mouse study [103].

**Figure 2:**
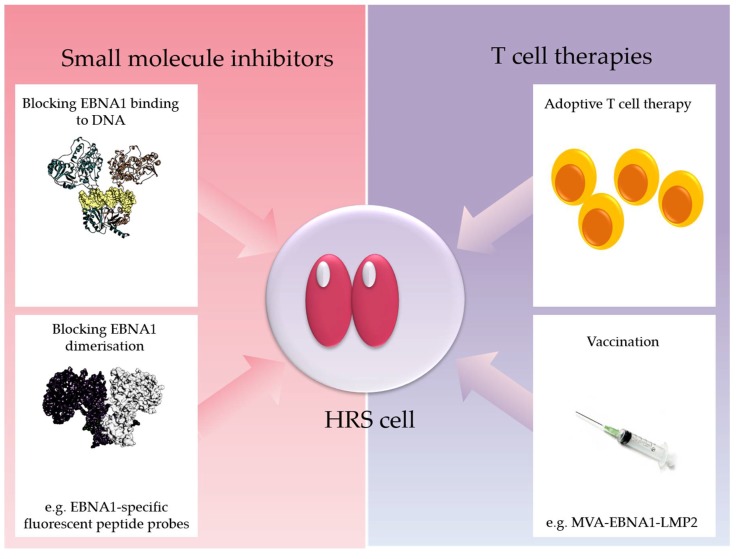
EBNA1 is an emerging target for therapeutic intervention. Shown is an HRS cell expressing EBNA1. Possibilities to target EBNA1 therapeutically are indicated and include the use of small-molecule inhibitors of EBNA1, for example using drugs that block EBNA1 binding to DNA and EBNA1-specific fluorescent peptide probes which prevent EBNA1 dimerization [109,110,111], therapeutic EBV vaccines including MVA-EBNA1-LMP2 containing an EBNA1-LMP2 fusion protein [112], and adoptive T-cell therapies [113,114]. Other approaches include relieving Gar-mediated suppression of EBNA1 translation which can potentially boost EBNA1 recognition by T cells (e.g., PhenDC3) [115].

EBNA1 could mediate some of its effects on cellular transcription through its bipartite Gly–Arg-rich domain which resembles the AT-hook of High Mobility Group A architectural transcription factors. This domain of EBNA1 mediates an interaction with cellular chromatin causing increased mobility of histone H1 [104]. EBNA1 also interacts with numerous sequence-specific host chromosome sites through its C-terminal DNA-binding domain [105,106]. Recently, it was shown that multiple EBNA1 binding sites are located proximal to transcription start sites in the human genome [107,108]. In one of these studies, EBNA1 depletion from LCL reduced their proliferation and led to the loss of expression of cellular genes that were also shown to bind EBNA1 in ChIPseq experiments [108]. These included MEF2B, EBF1 and IL6R, which, when depleted, partially phenocopied EBNA1 depletion by decreasing the cell growth and viability of latently infected cells [108]. Thus, EBNA1 is apparently capable of regulating key survival genes in B cells. Because of the potential critical role of EBNA1 in maintaining virus infection and also potentially in driving oncogenesis, there is emerging interest in targeting EBNA1 therapeutically (Figure 2).

#### 3.4.2. Latent Membrane Protein-1 (LMP1)

LMP1 has many functional similarities to a constitutively activated CD40 receptor [8,116,117,118,119,120], and is able to induce cell signalling pathways relevant to HL pathogenesis including the NF-κB, JAK/STAT, AP-1 and phosphatidylinositol-3 kinase (PI3K)/AKT pathways described earlier [120,121,122,123,124]. In fact, LMP1 could provide the signal for the activation of these pathways in the absence of mutations. This is well illustrated in the case of the TNFAIP3 gene which encodes a protein that is a negative regulator of NF-κB, and is almost always mutated only in EBV-negative cHL [125,126].

Because it regulates multiple signalling pathways, LMP1 is a major EBV regulator of cellular transcription. LMP1 can modify cellular transcription through a multitude of mechanisms that include the overexpression of the transcription factor ID2 [127], through DNA methyltransferases and protein arginine methyltransferases [128,129,130], by modification of the H3K27me3 histone mark, or alternatively by changing the levels of cellular miRNAs [131,132,133,134,135,136]. c-FLIP, which negatively regulates Fas-induced apoptosis, is also induced by LMP1, and this way could contribute to the rescue of BCR-negative GC B-cell progenitor from apoptosis [137,138]. LMP1 also downregulates expression of the telomeric repeat binding factors, TRF1, TRF2 and protection of telomeres (POT)-1 [139,140]. This leads to 3D shelterin disruption, resulting in telomere dysfunction, development of complex chromosomal rearrangements, and the generation of multinucleated HRS-like cells. LMP1 can also inhibit the differentiation of EBV-infected B cells to the plasma cell stages by inhibiting the expression of BLIMP1α, potentially contributing to a block in differentiation at the HRS-like stage [141].

LMP1 probably also contributes to the formation of the cHL TME because it has been shown to induce secretion of multiple chemoattractants in EBV-infected HRS cells [142,143,144]. While the cHL TME can support HRS cell growth and survival, it also helps the tumour cells escape EBV-specific immunity, including CD4+ and/or CD8+ T cells which can recognise epitopes from the viral latent proteins expressed in HRS cells [145,146,147,148,149,150,151,152,153,154,155].

A major immune evasion mechanism in cHL involves the overexpression of the programmed death ligands, PD-L1 and PD-L2, which are encoded by the CD274 and PDCD1LG2 genes, respectively. PD-L1 is overexpressed by tumour cells and macrophages in both EBV-positive and EBV-negative cHL, and in EBV-associated DLBCL [156]. In contrast, neither the malignant nor the nonmalignant cells of NLPHL, DLBCL NOS (not otherwise specified), or BL, express PD-L1 [156]. LMP1 can induce PD-L1 expression [157]. Amplification of PD-L1 and PD-L2 or reciprocal translocation involving CIITA also contribute to their overexpression in cHL [158]. Co-expression of PD-1 and PD-L1 is associated with poor prognosis in cHL [159].

Virus gene expression in HRS cells can also be modified by the interaction of EBV-infected tumour cells with the cHL TME. This is well illustrated in the case of LMP1, the expression of which can be stimulated by exposure of HRS cells to cytokines such as IL-4, IL-10, IL-13 and IL-21 [143,160,161]. The cHL TME can also potentially influence the outcome of virus gene expression. As described above, the collagen receptor DDR1 is overexpressed in cHL and we have shown that this effect is the result of its upregulation by LMP1 [71]. Thus, LMP1 can promote DDR1 activation and the survival of HRS cells, but only when collagen is present in the cHL TME [71].

#### 3.4.3. Latent Membrane Protein-2 (LMP2)

There are two LMP2 isoforms, LMP2A and LMP2B. They differ insofar as the 5′ exon of LMP2B is noncoding. LMP2A functions as a BCR mimic, allowing B-cell development in the absence of normal BCR signalling [9,162]. LMP2A activates cellular signalling required for B-cell survival, including the RAS/PI3K/AKT pathway [163]. EBV can immortalise BCR-negative GC B cells in vitro [164,165,166] and LMP2A is essential for this [167]. LMP2A can induce entry to the EBV lytic cycle in the absence of a functional BCR, but cannot do so when downstream BCR components are missing, as is the case in cHL [168]. LMP2A suppresses B-cell lineage gene expression, and is thus able to recapitulate some aspects of aberrant gene expression observed in HRS cells [168,169,170,171]. LMP2A constitutively activates Notch1 signalling which contributes to the loss of B-cell identity through altered transcription of E2A and EBF [172].

#### 3.4.4. Potential Interactions between LMP1 and LMP2A in B-Cell Lymphomagenesis

In transgenic mice, LMP2A expression induces autoimmunity [173], whereas LMP1 expression leads to B cell lymphoma [174,175]. However, the expression of both LMP1 and LMP2A in the same mouse B cells results in no significant B-cell abnormalities [176], suggesting that LMP2A may be a tumour suppressor. Recently, it was shown that LMP1 was dispensable for EBV-induced lymphoma formation in cord blood-humanized mice and that deletion of LMP2A delayed the onset of lymphoma in this model [177]. In another study, a mouse model was generated with conditional GC B-cell co-expression of LMP1 and LMP2A. There was little impact of LMP1 and LMP2A co-expression on the phenotype of B cells in immunocompetent mice [178]. However, when NK and T cells were depleted, there was extensive outgrowth of plasmablasts, characterised by overexpression of many markers known to be overexpressed in HRS cells, including CD30 [178].

## 4. Conclusions

EBV is associated with a variable subset of HL. Despite an increasing knowledge of the pathogenesis of these tumours, it is perhaps surprising that there are currently no standard-of-care therapies that target the virus or the molecular abnormalities specific to EBV-positive tumours. However, EBV-targeted therapies are in clinical development and in the near future could well be added to the increasing armory of drugs already available to HL patients. They include small-molecule inhibitors of EBNA1, which have been shown to be effective against EBV-infected cells in preclinical models, as well as adoptive T-cell therapy and therapeutic vaccination. These latter approaches could be envisaged to be particularly effective when used in combination with existing immune checkpoint therapies.
